# Elucidating the Antimycobacterial Mechanism of Action of Ciprofloxacin Using Metabolomics

**DOI:** 10.3390/microorganisms9061158

**Published:** 2021-05-28

**Authors:** Kirsten E. Knoll, Zander Lindeque, Adetomiwa A. Adeniji, Carel B. Oosthuizen, Namrita Lall, Du Toit Loots

**Affiliations:** 1 Department of Human Metabolomics, North-West University, Private Bag x6001, Box 269, Potchefstroom 2531, South Africa; kirsten.e.knoll@gmail.com (K.E.K.); zander.lindeque@nwu.ac.za (Z.L.); adenijiadetomiwa@gmail.com (A.A.A.); 2 Department of Plant and Soil Sciences, Faculty of Natural and Agricultural Sciences, University of Pretoria, Pretoria 0002, South Africa; carel.oosthuizen@uct.ac.za (C.B.O.); namrita.lall@up.ac.za (N.L.); 3 School of Natural Resources, University of Missouri, Columbia, MO 65211, USA

**Keywords:** fluoroquinolones, ciprofloxacin, untargeted metabolomics, *Mycobacterium tuberculosis*, tuberculosis, GCxGC-TOFMS

## Abstract

In the interest of developing more effective and safer anti-tuberculosis drugs, we used a GCxGC-TOF-MS metabolomics research approach to investigate and compare the metabolic profiles of *Mtb* in the presence and absence of ciprofloxacin. The metabolites that best describe the differences between the compared groups were identified as markers characterizing the changes induced by ciprofloxacin. Malic acid was ranked as the most significantly altered metabolite marker induced by ciprofloxacin, indicative of an inhibition of the tricarboxylic acid (TCA) and glyoxylate cycle of *Mtb*. The altered fatty acid, *myo*-inositol, and triacylglycerol metabolism seen in this group supports previous observations of ciprofloxacin action on the *Mtb* cell wall. Furthermore, the altered pentose phosphate intermediates, glycerol metabolism markers, glucose accumulation, as well as the reduction in the glucogenic amino acids specifically, indicate a flux toward DNA (as well as cell wall) repair, also supporting previous findings of DNA damage caused by ciprofloxacin. This study further provides insights useful for designing network whole-system strategies for the identification of possible modes of action of various drugs and possibly adaptations by *Mtb* resulting in resistance.

## 1. Introduction

Tuberculosis (TB), caused by *Mycobacterium tuberculosis* (*Mtb*), remains one of the leading causes of death globally from a single infectious agent [1], resulting in a mortality rate of 1.5 million and an infection rate of about 10 million annually [2]. Furthermore, the prevalence of drug resistant TB is also increasing, primarily due to poor adherence to the drug regimen in patients [3], as a result of the many side effects experienced by patients being treated with first-line anti TB medication, accompanied by the long treatment duration required [4,5]. Further contributing factors to developing drug resistant TB include inaccurate diagnosis, unsupervised treatment, poor economic status [6], and a further exacerbation in 2020 by the COVID-19 pandemic [2,7]. Currently, the WHO’s approved first-line therapy for patients with active TB is a 6-month “directly observed treatment short-course” (DOTS) regimen consisting of isoniazid (INH), ethambutol (EMB), pyrazinamide (PZA), and rifampicin (RIF) [8,9]. Infection with multi-drug resistant (MDR)-TB and extensively drug resistant (XDR)-TB requires treatment using various second-line antibiotics that are expensive, have far more side effects due to their higher toxicity, and need to be consumed for even a longer duration [2,10]. The only newly approved drugs for TB over the past 50 years are the second-line drugs for treating MDR-TB—linezolid, bedaquiline and delamanid—but not long after, resistance followed [9,11,12]. Considering this, there is an urgent need for well-tolerated and effective treatments for TB using drugs with novel modes of action against the infectious organism. 

A suggested approach for avoiding the long drug trial phases usually required for approving new drug candidates is further investigation of already existing drugs, repurposed for use in treating TB/MDR-TB, [13,14]. In order for a drug to be selected for possible repurposing applications, it should preferably be affordable, easily available, and show good pharmacokinetic/pharmacodynamic properties. For these reasons, several fluoroquinolones (FQs) are being intensively investigated for use as anti-TB therapy [15]. FQs, originally used to treat urinary tract infection [16], were first shown to be effective against *Mtb* in 1984, and have since gained continuous interest for such applications [17,18,19]. Currently, they are among the most frequently prescribed drugs [20] and are considered the backbone of MDR-TB treatment [10,21]. FQs target two *Mtb* topoisomerase deoxyribonucleic acid (DNA) enzymes, DNA gyrase and topoisomerase IV [22]. The first introduces negative super helical twists in the bacterial DNA double helix and catalyzes the separation of daughter chromosomes [23], whereas the latter is responsible for the segregation into two daughter cells at the end of DNA replication [24]. Earlier generations, ciprofloxacin and levofloxacin, exhibit greater activity against Gram-negative bacteria (and some Gram-positive bacteria) and target mainly DNA gyrase [25,26,27]. FQs inhibit DNA gyrase by binding to the enzyme and DNA, which leads to double-stranded DNA breaks [28,29,30,31,32]. 

The innate resistance mechanism of *Mtb* to many anti-TB drugs can be attributed to its complex cell wall [33]; however, the specific, highly lipophilic characteristics of FQs [34] provide great permeability over this [35]. These antibiotics chelate with Mg^2+^ cations and electrostatically interact with membrane phosphodiesters, subsequently traversing the *Mtb* cell wall [36]. FQs are not exempt from resistance, however, of which the best described are mutations in genes *gyrA* and *gyrB,* encoding subunit GyrA and GyrB of DNA gyrase [37,38,39]. Furthermore, several resistance-forming proteins have also been identified, including the efflux pumps, LfrA [40,41], and MmpL (mycobacterial membrane protein large) [42], the target protection proteins, MfpA and MfpB (*Mycobacterium* fluoroquinolone resistance proteins A and B) [43,44], and the cell-survival promotor, HtrA2 (high temperature requirement A) [45]. DNA repair and mutations by the SOS regulon have also been described [18]. 

Although interactions between DNA gyrase and FQs have been thoroughly investigated [22,38,46], little is known about their biochemical mechanisms of action against *Mtb* specifically, or *Mtb* drug resistance to these [10,19]. The results published thus far are somewhat contradictory. In a study by Verma et al. [47], the macromolecular composition of the *M. smegmatis* cell wall after sub-MIC ciprofloxacin treatment indicated a significant decrease in the total lipids, phospholipids, and sugars, suggesting ciprofloxacin-induced alterations of the cell wall. In contrast, Halouska et al. [48] indicated ciprofloxacin-induced inhibition of transcription, translation, and DNA supercoiling, without changes to the cell wall.3 While most data suggest cell death due to the inhibition of DNA replication [49], altered DNA biosynthesis could set in motion secondary events contributing to ciprofloxacin’s bacteriostatic or bactericidal effects. Furthermore, it is important to remember that stronger target activity does not predict better antimycobacterial activity. This is perfectly demonstrated by ciprofloxacin dimers, which show enhanced DNA gyrase inhibition, while less effectively killing *Mtb* [50]. This is thought to be due to a stronger cleavage of FQ to the DNA-enzyme complex, which results in less single-strand DNA fragments, and subsequently prevents RecA from recognizing damaged DNA and inducing the SOS regulon [32]. The SOS response assists in killing by releasing ROS [51,52], yet simultaneously activates DNA repair and creates a dormancy state, ultimately leading to resistance. Before this SOS response can be used to advantage as a possible mode of action against *Mtb*, this phenomenon, and how it connects to the mechanism of ciprofloxacin, still needs to be elucidated.

The selection of ciprofloxacin as our investigational compound was predominantly based on its safety profile. Although less potent than moxifloxacin (MIC 0.12–0.5 μg/mL), and levofloxacin (MIC 1 μg/mL), ciprofloxacin (MIC 0.5–4.0 μg/mL) [53] has the lowest risk for causing serious ventricular arrhythmia, cardiovascular mortality, and hepatotoxicity [54,55]. Furthermore, ciprofloxacin demonstrates the highest clearance rate of all FQs [56] and is thus the preferred option for the treatment of renally impaired patients [17]. The levels of ciprofloxacin in cerebrospinal fluid can be as high as 40–90% compared to that in plasma [53], which offers further advantages for its use in the treatment of tuberculous meningitis. Adverse drug reactions (ADRs) are usually minimal (5% or less), and the most common ADRs are usually gastrointestinal in nature (nausea, vomiting, diarrhea, and abdominal pain) [57,58]. Previous studies demonstrated that mitochondrial topoisomerases bear less than 30% homology to their prokaryotic counterparts and are not inhibited [23,59], but it has been reported that ciprofloxacin does affect mitochondrial DNA synthesis [60]. The pharmacological advantages of ciprofloxacin have not gone unnoticed, as stated by the World Health Organization [61], which has included ciprofloxacin as a critically important antibiotic.

Most of the evidence brought to light thus far has been generated using genomics, transcriptomics, or proteomics [62]. Metabolomics, the latest addition to “omics” technologies, identifies the down-stream metabolites of altered pathways and therefor presents a more sensitive level of organization, from which up-stream deductions can be made [8,63]. We identified the metabolite markers best differentiating *Mtb* with and without ciprofloxacin, using a two-dimensional gas chromatography coupled with time-of-flight mass spectrometry (GCxGC-TOF-MS) metabolomics approach, combined with universally connected metabolic libraries and advanced statistical analysis, in order to better elucidate its mechanism of action.

## 2. Materials and Methods

### 2.1. Bacterial Culture

Antimycobacterial minimum inhibitory concentration (MIC) and sub-MIC (50% inhibitory concentrations (MIC_50_)) of ciprofloxacin were determined via the Alamar Blue assay [64]. The cell cultures (5 individually cultured samples per group) were prepared as previously described [65], in the presence and absence of ciprofloxacin. All reagents were purchased from Sigma-Aldrich, St. Louis, MO, USA, unless otherwise stated. Briefly, *Mtb* H37Rv ATCC 27294 (kindly obtained from the Medical Research Council, Pretoria, Gauteng, South Africa) was cultured and maintained for 4 weeks on Lowenstein Jensen (LJ) slants. The bacterial inoculum was prepared to a McFarland standard of 1 (approximately 3 × 10^8^ colony-forming units/mL) in Middlebrook 7H9 broth supplemented with 10% OADC (oleic acid, albumin, dextrose, catalase) (Becton, Dickinson, UK) and 2% PANTA (polymyxin B, amphotericin B, nalidixic acid, trimethoprim, and azlocillin) (Becton, Dickinson, UK). PANTA was added for the prevention of contamination with negligible impact on *Mtb’s* growth [66]. Ciprofloxacin was dissolved in DMSO (150 µM), added as a vehicle control, and diluted into Middlebrook 7H9 broth to a final concentration of 0.3 µM (0.12 µg/mL) (0.2% DMSO). One milliliter of the prepared inoculum was added to yield a final assay volume of 5 mL, with a bacterial test concentration of 6 × 10^7^ CFU per 1 mL of ciprofloxacin. For the untreated *Mtb* control samples, 4 mL of Middlebrook 7H9 broth (0.2% DMSO) was added to each replicate culture, followed by the addition of the bacterial inoculum as described above. The DMSO solvent was kept constant throughout the assay. After 5 days of incubation at 37 °C, the samples were centrifuged to pellet the bacteria at 4500 rpm for 15 min. The pellets were washed with 1 mL of PBS and pelleted again under the same conditions. Finally, the PBS was aspirated from the samples and the pellets were stored immediately at −80 °C until further testing.

### 2.2. Whole Metabolome Extraction Procedure and Derivatization

The metabolites were extracted from the samples and derivatized as previously described by Beukes, et al. [67], with slight modifications. Briefly, 8 mg of each of the individually cultured samples were weighed out into an Eppendorf tube, followed by the addition of 50 µL 3-phenylbutyric acid (0.13 mg/ml H_2_O) (Sigma-Aldrich, Lot#536478V) as internal standard. One milliliter of a chloroform: methanol: water (1:3:1 ratio) solution was added, after which the Eppendorf tubes were shaken in a vibration mill at 30 Hz for 5 min, with a 3 mm carbide tungsten bead in each. The samples were centrifuged at 12,000 rpm for 5 min and the supernatant was transferred to a GC glass vial. The extracts were dried under a nitrogen stream, followed by the addition of 50 µL methoxamine hydrochloride (Sigma-Aldrich, Lot#BCBP2843V) in pyridine (Lot#S2BC335SV) at a concentration of 15 mg/mL. The glass vials were heated at 50 °C for 90 min. Following, methoximation, 40 µL *N,O*-bis(trimethylsilyl)trifluoroacetamide with 1% trimethylsilyl chloride (Lot#BCBW2670) was added, and vials were heated again for 60 min at 50 °C. Each extract was then transferred to a 0.1 mL vial insert in a GC sample vial and injected into GCxGC-TOF-MS.

### 2.3. GCxGC-TOFMS Analysis

A 4D Pegasus GCxGC-TOF-MS (LECO Africa (Pty) Ltd., Johannesburg, South Africa) equipped with a Gerstel Multi-Purpose Sampler (Gerstel GmbH and Co. KG, Mülheim an der Ruhr, Germany) and an Agilent 7890 gas chromatograph (Agilent, Atlanta, USA) coupled to TOF-MS (LECO Africa) were used for the analysis. The samples were analyzed in random sequence, with split-less injection. To monitor the analytical performance throughout the entire analysis, a quality control (QC) sample was analyzed at regular intervals. The processed samples were injected into Rxi-5Sil MS primary capillary column (28.8 m × 0.25 mm internal diameter, 0.25 µm film thickness, Restec), and a Rxi-17 secondary capillary column (1.2 m × 0.25 mm internal diameter, 0.25 µm film thickness), for GC compound separation. The primary GC oven temperature was set at 70 °C for 2 min, and then increased at a rate of 4 °C/min to a final temperature of 300 °C, at which it was maintained for an additional 2 min. The secondary oven was set at 85 °C for 2 min, increased at 4.5 °C/min, to a final temperature of 300 °C, at which it was maintained for 4.5 min. Helium, set to a column flow rate of 1 mL/min, was used as a carrier gas, and held at a constant temperature of 270 °C. Mass spectrometric data acquisition was carried out at −70 eV, with a solution delay of 350 sec, and a mass range of 50–800 *m*/*z* was scanned with a rate of 200 spectra/sec.

### 2.4. Data Processing, Clean-Up, and Statistics

ChromaTof software (version 4.32) was used for mass spectral deconvolution (at a signal to noise ratio of 20), peak alignment, and peak identification on the obtained mass spectra. Metabolites were identified by comparing their mass fragment patterns to those of compounds in commercially available databases containing previously injected standards. For normalization and assessment of data quality, the data were pretreated using a standardized metabolomics data clean-up procedure [67]. Each detected compound was normalized using MS total useful signal (TUS), which is based on a factor calculated from the sum of all metabolites identified in all samples, and by calculating the relative concentration of each by using the internal standard. All missing/zero values were replaced by a value calculated as 20% of the minimum detection limit of the entire dataset, as these are most likely present in sub-minimum concentrations rather than being completely absent [68]. An 80% data filter was then applied to eliminate compounds with more than 80% zero values within both groups [69]. To provide a balanced representation of all metabolites, log transformation and auto-scaling (mean-centered and divided by the standard deviation of each variable) were applied. This prevents compounds with minor concentrations from being overlooked due to the domination of compounds with higher concentrations [70]. Making use of MetaboAnalyst (Version 5.0) [71], multivariate statistical methods in the form of unsupervised principal component analysis (PCA) and supervised partial least squares-discriminant analysis (PLS-DA) were applied [72]. Subsequently, uni-variate analysis was performed by calculating t-test and effect size values [73].

Relationships between the selected metabolites were mapped using the KEGG, MetaCyc, and BioCyc databases, in addition to intensive research of the previously published literature on the topic.

## 3. Results

### 3.1. Data Overview

**Figure 1:**
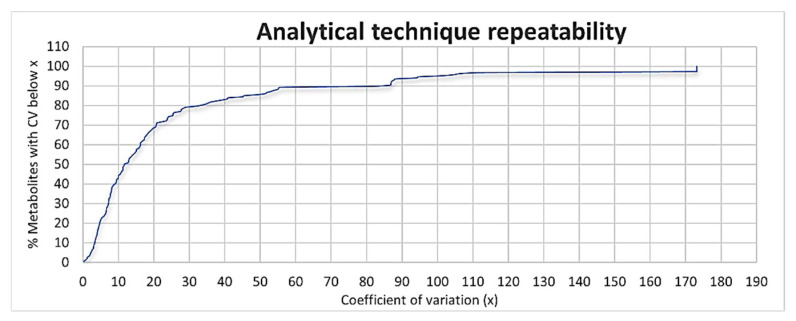
Distribution of the coefficients of variation values for technical repeatability.

**Figure 2:**
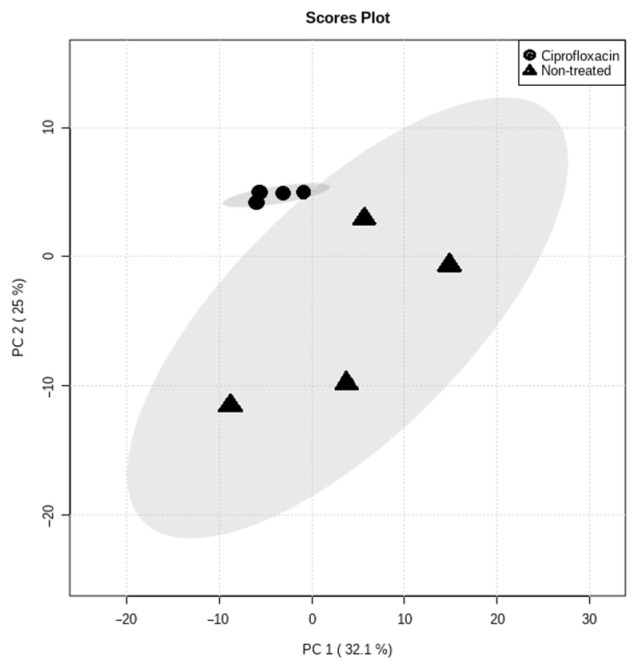
PCA scores plot obtained from GCxGC-TOFMS whole metabolome analysis of *Mtb* samples in the presence and absence of ciprofloxacin. The variances accounted for are indicated in parentheses.

When visualizing the analytical technique’s repeatability graphically (Figure 1), approximately 86% of all the compounds identified (*n* = 260) had a coefficient of variation (CV) value under 50%, while 70% had CV values under 20%. The analytical technique used during this analysis thus proved to be highly repeatable and can could be trusted to provide reliable results. PCA was initially used to obtain an overview of the natural grouping of metabolic data (Figure 2). The total variance between the groups, described by the first two principal components (PCs), was 57.1%, of which PC1 and PC2 accounted for 32.1% and 25%, respectively. The PCA scores plot of the metabolite data analyzed by GCxGC-TOF-MS shows clear clustering between *Mtb* in the presence and absence of ciprofloxacin, as represented in Figure 2. 

### 3.2. Marker Selection

**Figure 3:**
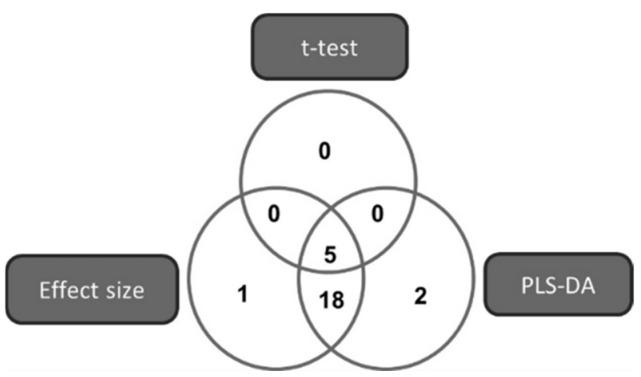
Venn diagram illustrating the multi-statistical approach for selecting the metabolites that best describe the variation detected in the metabolome of *Mtb* cultured with and without ciprofloxacin.

The metabolite markers (*n* = 26) best describing the differences between the ciprofloxacin and control samples were selected based on compliance with the following criteria: a PLS-DA VIP value > 1 [74], a *t*-test *p*-value < 0.05 [75], or an effect size > 0.8 [76] (Figure 3).

The selected metabolite markers are listed according to their PLS-DA VIP values in Table 1, along with their respective average concentrations and univariate test outcomes. Of the total, 61.5% (16/26) of the markers were elevated; most of these were fatty acids. The most differentiating marker was malic acid, with an exceptionally high d-value of 6.621 and low *p*-value of > 0.0001.

## 4. Discussion

**Figure 4:**
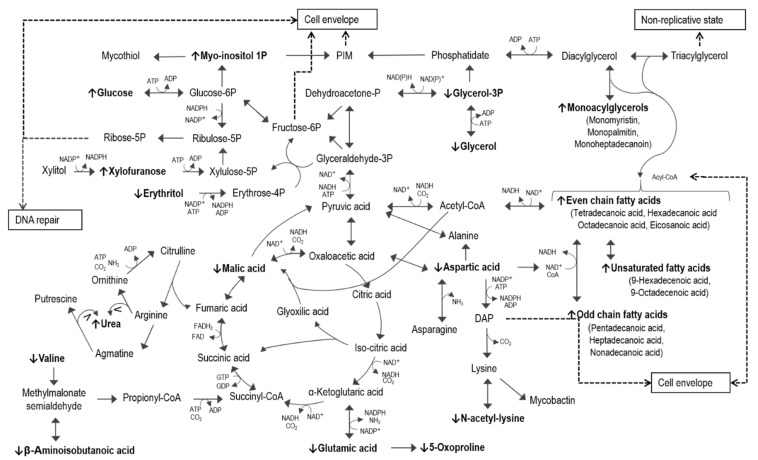
Metabolomic pathway map of *Mtb* treated with ciprofloxacin. The metabolite markers best describing the variation in the metabolome compared to those of untreated *Mtb* are represented in bold text with up or down arrows indicating elevated or reduced concentrations, respectively.

In this study, we identified a number of significantly altered metabolites induced by the administration of ciprofloxacin to *Mtb* culture, which when interpreted, in the light of known metabolism and previous ciprofloxacin findings, better elucidate its mechanisms of action against *Mtb*, as shown in Figure 4. The most prominently altered pathways included gluconeogenesis, fatty acid metabolism, amino acid metabolism, the pentose phosphate pathway (PPP), and the urea cycle.

Of note were the elevated levels of various even- and odd-chain saturated fatty acids of between 14 to 20 carbons (C14:0–C20:0) in length, in the *Mtb* treated with ciprofloxacin. This was also true for two Δ^9^-unsaturated fatty acids; 9-hexadecenoic (Δ^9^C16:1), and 9-octadecenoic (Δ^9^ C18:1) acids. These indicate a strongly upregulated synthesis toward cell wall repair, supporting previous evidence associating ciprofloxacin with cell wall damage [47]. Simplified, lying outside of the cytoplasmic membrane, a peptidoglycan (PG) layer is covalently attached to arabinogalactan (AG), which itself attaches to mycolic acids (MA) to form the MA-AG-PG complex (MAPc) [77,78]. Interspersed within the MAPc, are the glycerolipids, phosphatidyl myo-inositol mannosides (PIM) and lipoarabinomannans (LAM) [79]. PIM is a crucial part of the membrane structure and serves as a precursor of LAM [80]. The saturated fatty acid markers in this study are produced by fatty acid synthase type I (FAS I). FAS I generates 16 to 26 carbon length fatty acyl-coenzyme As (CoA) [81], which are fed into FAS II for elongation. FAS I and FAS II provide acyl groups for the synthesis of all cell envelope components, except for AG [82,83]. Δ^9^C16:1 and Δ^9^ C18:1 and their precursors, hexadecanoic (C16:0) and octadecanoic (C18:0) acid, respectively, are considered major fatty acids of glycerolipids and mycolic acids [84,85,86]. Δ^9^C16:1 and Δ^9^ C18:1 are reduced from C16:0 and C18:0, in the presence of Fe^2+^, a flavin, NADPH, and O_2_ [87,88]. Interestingly, some *mmpL* genes, encoding fatty acid transporter protein MmpL have been shown to be repressed when their transcriptional regulator proteins bind to C:16 fatty acids and monoacylglycerols (MAG) [89,90]. Even so, further research is needed to establish possible activity of different fatty acids on different MmpL regulator proteins. It is, however, important to note that the damage that ciprofloxacin administration induces to the cell wall may be direct, but is most likely indirect, by inhibition of other energy-producing mechanisms or simply by induction of the SOS response in *Mtb*, shifting energy production away from glucose toward the preferential use of fatty acids [91]; hence, less of these fatty acids are now available to cell wall synthesis. Increased FA synthesis or the accumulation of cell wall components has also been identified in *Mtb* treated with EMB, INH, PZA, RIF, and pretomanid [92,93,94,95,96].

The dramatically elevated synthesis of intracellular fatty acids would be expected to consume a considerable amount of carbon, which can be supplied from various sources [91]. However, as will be explained, glucose and glycerol seem to be the major suppliers for such, and for the various components required for DNA repair. This is supported by previous findings indicating ROS, produced during the FQ-induced SOS-response, causes oxidative stress, which in turn activates utilization of triacylglycerol (TAG) and cell wall lipids for energy [97,98,99], as is generally the case during the non-replicative phase of *Mtb* [100,101]. These results are also supported by previous findings showing reduced concentrations of phospholipids and mycolic acids in the cell wall macromolecules of sub-MIC ciprofloxacin-treated *M. smegmatis* [47], because these are now being preferentially used for energy production, with glucose supplying the necessary carbon substrates for the continued synthesis of these much-needed fatty acids, which are now preferentially used for energy production.

A shift in energy supply to β-oxidation of fatty acids, as opposed to the TCA cycle, reserves NAD^+^, in addition to CO_2_, for *de novo* synthesis of nucleotides (for DNA repair and fatty acids). NADH and NADPH released during fatty acid metabolism fuel the upregulated non-oxidative PPP and glycerolipid metabolic pathway. In this study, the downregulated TCA cycle was indicated by a reduction in malic acid and aspartic acid, which according to the uni- and multivariate statistics, were ranked as the two most important metabolite markers altered by ciprofloxacin (Table 1). Aspartic acid is considered a validated reporter of oxaloacetate (OAA) [102], and together with malic acid, supports TCA cycle inhibition. Although oxidative stress is normally associated with an increase in glyoxylate shunt activity, in this study the glyoxylate shunt was clearly downregulated. This indicates a greater need of carbon flux through gluconeogenesis toward the PPP and glucose, for subsequent fatty acid and nucleotide synthesis [103,104]. Reduced aspartic acid concentrations were also previously found in a study investigating phenotypic antibacterial persistence in *Mtb* [105]. These results support the SOS response-induced decrease in oxidative phosphorylation and increase in energy reserves for DNA repair in response to FQs [32,102]. It is noteworthy to mention that the ATPase activity of DNA gyrase (29), shows reduced adenosine triphosphate (ATP) conversion in the presence of ciprofloxacin [106], which most likely is for the purpose of reserving ATP for other energy consuming pathways, such as gluconeogenesis and DNA repair [107].

Various PPP intermediates that contribute to DNA and cell wall synthesis were detected to be altered in the ciprofloxacin-treated *Mtb*, [99,108,109]. Under normal circumstances, erythrose-4-phosphate and glyceraldehyde-3-phosphate produce xylulose-5-phosphate (xylulose-5P) and fructose-6-phosphate (fructose-6P) [91]. The latter is converted to glucose-6-phosphate, for subsequent PIM synthesis, via the myo-inositol pathway [110], and to glucose-*N*-acyl 6-phosphate (GlcN6P), for subsequent PG synthesis [111]. Xylose-5P is the precursor of ribose-5-phosphate (ribose-5P), which, in the presence of ATP, is converted to 5-phosphoribosyl-1-pyrophosphate (pRpp) [103,112]. PRpp is the branch point intermediate of decaprenyl-phospho-arabinofuranose (DPA) [113], the only donor of arabinose to AG and LAM [104,114] and nucleotide synthesis. In this investigation, the upregulated non-oxidative PPP in the ciprofloxacin-treated *Mtb* was supported by the elevated concentrations of xylofuranose and reduced erythritol (Figure 4). Furthermore, it is well known that PPP metabolism is fueled by glucose and the glucogenic amino acids valine, aspartic acid, and glutamic acid [115], all of which were specifically and significantly reduced in the ciprofloxacin-treated *Mtb*, in addition to their degradation products β-aminoisobutanoic acid [116], 5-oxoproline [117], and N-acetyl-lysine [118], respectively (Figure 4). Furthermore, the reduced levels of glutamic acid and aspartic acid in our investigation supported an oxidative state in the ciprofloxacin-treated group [119]. The above-mentioned amino acids also serve as precursors of cell wall related intermediates. Alanine derived from aspartic acid combines with valine to produce CoA, which is used for converting fatty acids into cell wall lipids [120,121,122]. Valine also serves as a precursor of propionyl-CoA [116,123] and is used for the elongation of odd-chain fatty acids [124]. Aspartic acid additionally is the precursor of NAD^+^, 2,6-diaminopimelate (DAP) [125,126], and S-adenosyl methionine (SAM) [127]. Considering this, NAD^+^ is subsequently an important cofactor for FAS I [128], and SAM is required for the methylation of cell-wall fatty acids [129]. Furthermore, the aforementioned DAP, along with alanine and glutamic acid, serve as substrates for PG [130]. Lastly, the observed flux through aspartic acid can be supported by the elevated levels of urea in *Mtb* treated with ciprofloxacin [131], and considering that aspartic acid is one of the top three metabolite markers identified (Table 1), it is possibly utilized, contributing to many of the cell-envelope changes observed.

The FQ-induced SOS response is known to cause cell growth arrest [132], and in this study, inhibited cell growth is evident by the increased TAG metabolism and reduced TCA activity, in addition to the inhibited protein synthesis observed in the ciprofloxacin-treated *Mtb*. Protein synthesis requires ATP-dependent activation of glutamate and aspartate, followed by amination by ammonia, to form aminoacyl-tRNA [115]. However, in this study, urea was found to be elevated in *Mtb* treated with ciprofloxacin, indicating accumulation of ammonia and reduced recycling of nitrogen via glutamine and proline metabolism. This, in addition to a decreased degradation of urea by urease, also linked to the *Mtb* stress response [133], retains ammonia from protein synthesis [134]. Inhibited protein formation has also been suggested in a proteomics study of *Mtb* treated with ciprofloxacin [135], as well as a metabolomics study of *M. smegmatis* treated with ciprofloxacin [48]. Disruption of protein synthesis has also been reported in studies investigating the response of *Mtb* to pretomanid, streptomycin, EMB, RIF, PZA, and INH [95,136,137,138]. The inhibited protein synthesis observed would in turn disrupt the functionality of membrane proteins, subsequently inhibiting nutrient uptake as well as fatty acid transport to the cell wall [42]. Additionally, urea acts as an osmolyte, preventing dehydration or water loss as a result of the seemingly damaged cell wall [139]. Increased urea could also be indicative of polyamine synthesis (Figure 4) [140]. Polyamines, such as putrescine, have been reported to reduce accumulation of trans-membrane proteins [141] and contribute to the phenotypic drug resistance to FQs [142]. 

Observed changes to the monoacylglycerols (MAG) 1-monomyristin, 2-monopalmitin, and 1-monoheptadecanoin are indicative of cell envelope changes via the glycerolipid and triacylglycerol (TAG) pathways. TAG in the cell wall [143] can be metabolized during the stress-induced transition to the non-replicated phase [144] and during infection [145]. *Mtb*’s adaptation during these circumstances involves the use of lipids as main energy reserves, as previously mentioned [146]. During the deacetylation of TAG to diacylglycerol (DAG), and from DAG to MAG, acyl-CoAs are released, which are either directed toward glycerolipid synthesis (Figure 4) or energy production [147,148]. Glycerolipid and TAG metabolism share an important intermediate at the branch point, 1,2-diacyl-*sn*-glycerol 3-phosphate, commonly known as phosphatidate (PA) (Figure 4) [149]. PA is synthesized via two pathways: 1) the phosphorylation of DAG by DAG kinase [150] or 2) the acylation of glycerol-3-phosphate (glycerol-3P) by glycerol phosphate and acyl glycerol phosphate acyltransferase [151]. In this study, the concentrations of glycerol-3-phosphate and its precursor glycerol were decreased, supporting a MAG metabolic flux toward TAG and PIM in the ciprofloxacin-treated *Mtb* group. Glycerol-3P appears to recycle the transport of membrane lipids [152] and can thus be expected to be the rate-limiting step, which would explain the accumulation of the fatty acids and the accompanying reduction in glycerol levels. The synthesis of glycerolipids also requires myo-inositol, which is produced via glucose-6-phosphate (glucose-6P) [153]. Firstly, myo-inositol 3-phosphate synthase converts glucose-6P into myo-inositol 3-phosphate, which in turn is dephosphorylated by several myo-inositol monophosphates to produce myo-inositol [84]. The elevated levels of myo-inositol monophosphates in the ciprofloxacin-treated *Mtb* hence further support the aforementioned flux toward glycerolipids [154]. Furthermore, myo-inositol-1-phosphate is converted by a glycosyltransferase, mycothiol (Msh). Mycothiol is also considered an important antioxidant required for balancing the cytosolic NAD^+^/NADH ratio [155]. In addition to the abovementioned inhibition of protein synthesis and enhanced fatty acid synthesis, altered energy metabolism has also consistently been identified in response to the first-line drugs INH [156], EMB [157], PZA [158], and RIF [159], supporting these results. In summary, the mycobacterial cell wall metabolism is visibly linked to the SOS response, which has frequently been proposed to cause resistance toward ciprofloxacin and challenge the otherwise impressive bactericidal activity of this drug [18,160,161,162].

## 5. Conclusions

In this study, we investigated the metabolic changes to *Mtb* induced by sub-MIC of ciprofloxacin, in order to better understand its mechanism of action and the resultant adaptations of *Mtb*. Previous studies have identified alterations in transcription, translation, and cell wall synthesis as part of the mechanism of action of ciprofloxacin against *Mtb* (51, 52, 135). Our metabolomics study identified metabolite markers that support previous results, as indicated by the drastic accumulation of metabolites associated with cell wall and DNA repair. Moreover, many of these markers indicate an SOS-induced shift to the non-replicative phase, which is a key mechanism determining *Mtb* persistence and tolerance to various anti-TB drugs. This study not only gives a better understanding of ciprofloxacin’s mode of action but provides helpful insight for further investigation of antibiotic-induced resistance by *Mtb*, and also perhaps the use of ciprofloxacin in combination with existing anti-TB drugs. 

## Figures and Tables

**Table 1:** Metabolite markers best describing the variance between the individually cultured *Mtb* samples in the absence (*Mtb* controls) and presence of ciprofloxacin.

Metabolite Name (ChEBI ID)	Average Concentration (mg/g Cell Mass) (Standard Deviation)		*t*-Test (*p*-Value)	Effect Size (d-Value)	PLS-DA (VIP)	Fold Change (log_2_)
	*Mtb* with Ciprofloxacin	*Mtb* Controls				
Malic acid (6650)	0.033 (0.002)	0.054 (0.004)	0.000	6.621	2.012	−0.39
Aspartic acid (17053)	0.007 (0.001)	0.013 (0.004)	0.008	2.236	1.732	−0.46
Glycerol (17754)	0.497 (0.033)	0.633 (0.031)	0.013	1.932	1.678	−0.21
5-Oxoproline (17203)	0.069 (0.014)	0.099 (0.020)	0.014	1.962	1.665	−0.30
Xylofuranose (46432)	0.075 (0.037)	0.034 (0.008)	0.037	1.376	1.511	1.21
Myo-inositol-1-phosphate (18297)	0.004 (0.001)	0.003 (0.001)	0.051	1.637	1.445	0.33
9-Hexadecenoic acid (28716)	0.005 (0.000)	0.004 (0.001)	0.091	1.329	1.299	0.25
Nonadecanoic acid	0.067 (0.011)	0.055 (0.008)	0.094	1.151	1.292	0.22
Heptadecanoic acid (32365)	0.015 (0.003)	0.011 (0.002)	0.095	1.355	1.288	0.36
Octadecanoic acid (28842)	0.582 (0.164)	0.371 (0.141)	0.096	1.241	1.287	0.57
Valine (16414)	0.017 (0.004)	0.022 (0.005)	0.106	1.117	1.255	−0.23
β-Aminoisobutanoic acid (33094)	0.011 (0.002)	0.014 (0.004)	0.121	1.256	1.216	−0.21
Glutamic acid (16015)	0.010 (0.001)	0.015 (0.006)	0.124	0.960	1.209	−0.33
Hexadecanoic acid (15756)	0.575 (0.051)	0.480 (0.077)	0.128	0.969	1.198	0.20
9-Octadecenoic acid (36021)	0.679 (0.060)	0.600 (0.041)	0.132	1.089	1.187	0.13
Tetradecanoic acid (28875)	0.089 (0.010)	0.071 (0.017)	0.141	0.934	1.167	0.25
Eicosanoic acid (28822)	0.005 (0.001)	0.004 (0.000)	0.146	0.991	1.154	0.25
Glucose (17234)	0.045 (0.006)	0.037 (0.005)	0.152	0.934	1.140	0.22
Urea (16199)	0.005 (0.002)	0.002 (0.002)	0.171	0.816	1.099	1.50
*N*-Acetyl-l-Lysine (64859)	0.002 (0.001)	0.004 (0.003)	0.175	0.884	1.090	−0.50
2-Monopalmitin (75455)	0.011 (0.002)	0.009 (0.001)	0.175	0.907	1.089	0.22
1-Monomyristin (75562)	0.007 (0.003)	0.005 (0.001)	0.175	0.882	1.089	0.40
Pentadecanoic acid (42504)	0.005 (0.001)	0.004 (0.001)	0.181	1.021	1.077	0.25
Glycerol 3-phosphate (15978)	0.005 (0.001)	0.007 (0.002)	0.186	0.908	1.067	−0.29
1-Monoheptadecanoin (144339)	0.010 (0.002)	0.007 (0.001)	0.186	0.947	1.066	0.43
Erythritol (17113)	0.021 (0.002)	0.023 (0.001)	0.202	0.856	1.034	−0.09

## Data Availability

Not applicable.
